# Association between the stage of labour during caesarean delivery with adverse maternal and neonatal outcomes among referred mothers to tertiary centres in resource-limited settings

**DOI:** 10.1136/bmjopen-2023-077265

**Published:** 2023-11-28

**Authors:** Dereje Zewdu, Temesgen Tantu, Fikretsion Degemu, Mukerem Abdlwehab

**Affiliations:** 1 Department of Anesthesia, Wolkite University, Welkite, Ethiopia; 2 Obstetrics and Gynecology, Wolkite University, Welkite, Ethiopia; 3 Department of Pediatrics and Child Health, Wolkite University, Welkite, Ethiopia

**Keywords:** OBSTETRICS, Anaesthesia in obstetrics, NEONATOLOGY

## Abstract

**Objective:**

Although the caesarean delivery (CD) rate has substantially increased, little is known about its impacts when performed in the first and second stages of labour on fetomaternal outcomes, especially among referred mothers. Thus, this study aimed to investigate the association between CDs performed during the first and second stages of labour and poor maternal and neonatal outcomes among mothers referred to tertiary centres.

**Setting:**

This retrospective cohort study analysed medical records of mother–infant pairs from September 2020 to May 2023 in Southern Ethiopia.

**Participants:**

We retrospectively collected data from 848 participants who underwent emergency CD on a referral basis during the study period.

**Primary outcome measure:**

The primary outcomes of interest were adverse maternal and neonatal outcomes. Data were analysed using descriptive and inferential statistics.

**Results:**

Of the 848 CDs, 722 (85.2%) and 126 (14.8%) were performed at the first and second stages of labour, respectively. Caesarean sections performed at the second stage were higher with nulliparity, increased maternal age, and birth weight. Compared with the first-stage CD, the second-stage CD was associated with a significantly increased risk of adverse maternal (OR 3.7, 95% CI 2.4 to 5.7) and neonatal outcomes (OR 2.0; 95% CI 1.3 to 2.9), including neonatal death.

**Conclusion:**

Second-stage CDs have an increased risk of adverse maternal and neonatal outcomes. Strengthening and improving obstetric emergency surgical services and intensive neonatal care for those populations would help decrease the maternal and fetal negative consequences.

**STRENGTHS AND LIMITATIONS OF THIS STUDY**

- The study evaluated the impact of second-stage caesarean delivery on fetomaternal outcomes among referred mothers using a larger sample size.
- The study followed the maternal and neonatal characteristics from admission to hospital discharge.
- The study included 3-year data, which is relatively representative of the existing problems.
- The retrospective nature of the study design may result in variable selection bias.
- Extrapolating the findings to non-referred participants might be difficult.

## Introduction

The caesarean delivery (CD) rate has significantly increased globally, representing one-third of total institutional deliveries in Ethiopia.^1 2^ Although CD is generally a life-saving procedure, it may lead to increased maternal and neonatal complications and healthcare expenses.^3 4^ In many low-income countries where equitable access to obstetric surgical services is limited, the patient frequently gets referred to nearby tertiary centres and receives delayed treatment, resulting in compromised maternal and neonatal outcomes.^5 6^

It is interesting to note that increased access to CD in resource-limited settings could help reduce the incidence of maternal and newborn morbidity and mortality.^7^ Nonetheless, being referred to other centres for CD and delays in timely intervention contribute to the likelihood of unfavourable fetomaternal outcomes.^8^ Despite these, the impact of the stage of labour during CD on maternal and neonatal adverse outcomes among those populations has not been well established. While clinicians carried out many CD procedures during the first stage of labour with fewer undesirable outcomes for both the mother and the newborn, the second-stage CD is technically more difficult and poses serious consequences.^9 10^

Previous studies demonstrated inconclusive and conflicting results on the association between CD at the stage of labour and fetomaternal outcomes. Some studies have found a significant correlation between CD performed at the second stage of labour and adverse maternal effects.^11–18^ On the contrary, others found no association between second-stage CD and poor maternal outcomes.^19–21^ Poor maternal outcomes among CD performed at the second stage of labour include increased blood loss, uterine atony, need for blood transfusion and perioperative complications.

Concerning neonatal outcomes, one study reported serious neonatal complications are common in the CD performed at the first stage of labour.^10^ Nevertheless, other studies found higher poor outcomes in second-stage CD^13 14 21 22^ and no association between these measurements.^15 17 23–25^ Neonatal adverse outcomes include a low fifth min Apgar score, increased need for neonatal intensive care unit (NICU) admission and neonatal death.

As far as our search, no other studies have compared the association between CD performed at the first and second stage of labour on their neonatal and maternal outcomes among referred mothers to tertiary centres. For practitioners to pinpoint the existing clinical gap and use the findings to improve clinical practice, it would be beneficial to understand the connections between CD carried out at the first and second stages of labour and undesirable newborn and mother outcomes.

Therefore, we aimed to investigate the association between CD performed at the first and second stage of labour with adverse maternal and neonatal outcomes among mothers referred to Wolkite University Referral and Teaching Hospital in Southern Ethiopia. The findings of this study provide information for clinicians, researchers and policy-makers to maximise the quality of perioperative obstetric and intensive neonatal care and decrease second-stage CD-related fetomaternal complications.

## Methods

### Study population and study design

This retrospective cohort study included all consecutive women who underwent primary CD for singleton term pregnancy between September 2020 and May 2023 at Wolkite University Specialized and Teaching Hospital in Ethiopia.

We included a total of 848 consecutive primary CD performed for referred mothers during the study period for final analysis. Exclusion criteria were as follows: mothers with multiple gestation, preterm birth, intrauterine growth restriction, fetal anomalies and incomplete documentation.

### Data collection

We retrospectively collected data from the maternal and neonatal medical records, including maternal and neonatal baseline characteristics, perioperative data, and adverse maternal and neonatal outcomes of all study participants using a checklist adapted from prior studies.^26^

The maternal and neonatal baseline characteristics included maternal age, body mass index, parity, residency area, educational status, onset and duration of labour, gestational age, birth weight and fetal sex. Perioperative maternal and neonatal characteristics included skin incision to delivery time, operation duration, estimated blood loss, uterine atony, unintentional uterine tear extension, uterine artery ligation, need for blood transfusion, ureteral/bowel injury, perioperative hysterectomy, need for intensive care unit (ICU) admission and maternal death, as well as fifth min Apgar score, need for NICU admission and neonatal death.

### Study outcomes

The primary outcomes of interest were adverse maternal and neonatal outcomes. The maternal outcomes included one or more of the following: perioperative haemorrhage, uterine artery ligation, unintentional uterine extension, ureteral or bowel injury, peripartum hysterectomy and maternal death. Neonatal adverse outcomes included one or more of the following: fifth min Apgar score <7, need for NICU admission and neonatal death.

### Statistical analysis

We entered, coded and analysed the data using SPSS (SPSS for Windows V.26.0, SPSS). We used the Student’s t-test test for numerical variables and described them as mean±SD.

We presented the categorical data as frequency and percentage, and the association between independent and dependent variables was analysed using the χ^2^ and Fisher’s exact tests for statistical differences between groups. Logistic regression analysis examined the impacts of CD performed at the first and second stages of labour on maternal and neonatal adverse outcomes. Results displayed as an OR with a 95% CI and a p<0.05 are considered statistically significant.

### Patient and public involvement

None.

## Results

### Maternal and neonatal baseline characteristics

Of 897 women who underwent primary caesarean section performed during the study period, about 848 (94.5%) were included for final analysis. Of these, 722 (85.1%) CDs were in the first stage and 126 ((14.9%) in the second stage of labour. While education, referral status, the labour onset and fetal sex between CD performed at the first and second stages were comparable, nulliparity, the onset of labour to delivery time, and birth weight were significantly higher in the second-stage CD 58.7% vs 35.7%; p<0.001), (14.8±10.4 vs 10.6±7.1; p<0.001), and (3298.1±338.3 vs 3444.1±322.3; p<0.001), respectively, as shown (table 1).

**Table 1:** Maternal and neonatal baseline characteristics between CD performed at the first and second stages of labour

Variables	CD at first stage of labour (n=722)	CD at second stage of labour (n=126)	P value
Maternal age, year, mean±SD	26.5±4.8	24.9±4.5	**<0.001**
BMI, Kg/m^2^, mean±SD	27.9±2.5	28.6±2.5	0.004
Nulliparity, n (%)			**<0.001**
Yes	258 (35.7)	74 (58.7)	
No	464 (64.3)	52 (41.3)	
Educational status, n (%)			0.17
Literate	588 (81.4)	96 (76.2)	
Illiterate	134 (18.6)	30 (23.8)	
Referral status, n (%)			0.62
Rural	338 (46.8)	62 (49.2)	
Urban	384 (53.2)	64 (50.8)	
The onset of labour, n (%)			0.65
Spontaneous	662 (91.7)	114 (90.5)	
Induced	60 (8.3)	12 (9.5)	
Labour onset to delivery, hours, mean±SD	10.6±7.1	14.8±10.4	**<0.001**
Neonatal			
GA at delivery, weeks, mean±SD	38.6±1.3	38.7±1.5	0.54
Birth weight at delivery, gram, mean±SD	3298.1±338.3	3444.1±322.3	**<0.001**
Fetal sex, n (%)			0.9
Male	375 (51.9)	66 52.4)	
Female	347 (48.1)	60 (47.6)	

A p<0.05 is considered significant.

The values in bold are statistically highly significant.

BMI, body mass index; CD, caesarean delivery; GA, gestational age.

### Perioperative maternal and neonatal characteristics

Maternal perioperative characteristics, as shown in (table 2), the CD performed at the second stage of labour was significantly associated with prolonged skin incision-to-delivery time, operation time, postpartum haemorrhage, uterine atony, uterine artery ligation, need for blood transfusion, unintentional uterine tear extension, perioperative hysterectomy, need for ICU admission and even ureteral/bowel injury (p<0.001, p<0.001, p<0.001, p<0.001, p=0.002, p=0.008, p<0.001, p=0.016, p=0.026, p=0.036, respectively).

**Table 2:** Perioperative maternal and neonatal characteristics between CD performed at the first and second stages of labour

Variables	CD at the first stage of labour (n=722)	CD at the second stage of labour (n=126)	P value
Skin incision to delivery time, minutes	6.1±1.6	7.7±2.2	**<0.001**
Duration of operation, minutes	41.8±20.1	49.4±26.1	**<0.001**
Duration of operation >1 hour, n (%)			**<0.001**
Yes	122 (16.9)	42 (33.4)	
No	600 (83.1)	84 (66.6)	
Estimated blood loss, mL	464.5±342.2	599.2±539.2	**<0.001**
Perioperative haemorrhage (> 1000 mL), n (%)			**<0.001**
Yes	44 (6.1)	20 (15.9)	
No	678 (93.9)	106 (84.1)	
Uterine atony, n (%)			**<0.001**
Yes	34 (4.7)	18 (14.3)	
No	688 (95.3)	108 (85.7)	
Uterine artery ligation, n (%)	18 (2.5)	10 (7.9)	0.002
Blood transfusion, n (%)			0.008
Yes	22 (3)	10 (7.9)	
No	700 (97)	116 (92.1)	
Unintentional uterine tear extension, n (%)	4 (0.5)	7 (5.7)	**<0.001**
Perioperative hysterectomy, n (%)	8 (1.1)	5 (4.1)	0.016
ICU admission, n (%)	12 (1.6)	6 (4.9)	0.026
Ureteral/bowel injury, n (%)	4 (0.5)	3 (2.4)	0.036^*^
Maternal death, n (%)	3 (0.4)	2 (1.6)	0.08
Neonatal			
Fifth minute Apgar score <7, n (%)			**<0.001**
Yes	60 (8.3)	28 (22.2)	
No	662 (91.7)	98 (77.8)	
Need for NICU admission, n (%)			**<0.001**
Yes	178 (24.6)	44 (34.9)	
No	544 (75.4)	82 (65.1)	
Neonatal death, n (%)			**<0.001**
Yes	26 (3.6)	18 (14.3)	
No	696 (96.4)	108 (85.7)	

The values in bold are statistically highly significant.

*Fisher’s exact test.

CD, caesarean delivery; ICU, intensive care unit; NICU, neonatal intensive care unit.

Similarly, neonatal outcomes, including fifth min Apgar score <7, need for NICU admission and neonatal death, were significantly higher in CD performed at the second stage than in the first stage of labour (22.2% vs 8.3%; p<0.001, 34.9% vs 24.6%; p<0.001, 14.3% vs 3.6%; p<0.001), respectively, as shown in (table 2).

### Association between CD performed at the first and second stage of labour with adverse maternal and neonatal outcomes

Logistic regression analysis showed that the likelihood of developing adverse maternal outcomes after CD performed at the second stage of labour was 3.7 times (OR 3.7, 95% CI 2.4 to 5.7) higher than the first-stage CD. Similarly, second-stage CD increases the risk of developing adverse neonatal outcomes twofold (OR 2.0; 95% CI 1.3 to 2.9) compared with CD performed at the first stage of labour, as shown in table 3.

**Table 3:** Association of adverse maternal and neonatal outcomes with CD performed at the first and second stages of labour

Outcomes	CD at the first stage of labour (n=722)	CD at the second stage of labour (n=126)	ORs (95% CI)	P value
Adverse maternal outcomes				**<0.001**
Yes	88 (12.2)	43 (34.1)	3.7 (2.4 to 5.7)	
No	634 (87.8)	88 (65.9)	1	
Adverse neonatal outcomes				**<0.001**
Yes	198 (27.4)	54 (42.8)	2.0 (1.3 to 2.9)	
No	524 (72.6)	72 (57.2)	1	

The values in bold are statistically highly significant.

CD, caesarean delivery.

## Discussion

In this retrospective study involving 848 women who underwent emergency CD at a referral hospital in Southern Ethiopia, we compared maternal and neonatal adverse outcomes of CD performed during the first and second stages of labour. Our main findings were that CD performed at the second stage was associated with an increased risk of maternal and neonatal adverse outcomes compared with CD performed at the first stage of labour.

Increased adverse maternal outcomes included longer operation times, estimated blood loss or postpartum haemorrhage, uterine atony and uterine artery ligation procedures, suggesting that CD at the second stage might be technically challenging and associated with higher odds of complications.^9 10^ This is likely due to the engagement of the fetal head in the pelvis, the difficulty in delineating the bladder, and the larger infants that are perhaps more difficult to remove from the uterus.^11 12^

The increased amount of blood loss because of uterine atony and unintentional uterine extensions is frequent in the second stage CD, as revealed in the present and previous studies.^13 14^ Other studies also showed that unintended uterine extension was associated with longer operation time, more blood loss, postpartum anaemia, and an increased need for blood transfusion.^15 16^

We believe that the extended labour duration and more substantial uterine fatigue may be the reason for the increase in uterine atony incidence in the second stage CD.^17^ Our study also showed because of the increased amount of blood loss and uterine atony, an increased need for blood transfusion and uterine artery ligation procedures are more frequent in second-stage CD, in line with previous reports.^18^ Therefore, it is crucial to consider effective uterotonic agents, leveraging the expertise of experienced obstetricians and continual monitoring to lessen maternal morbidity in these scenarios.

Contradicting our findings, a few studies^19–21^ reported no association between second-stage CD and more blood loss, uterine atony, and other poor maternal outcomes. These discrepancies may occur because of the variations in the study population recruited for analysis (urgency of the surgery, indications for CD and delayed presentation in their referral status), fluctuations in sample size and study design, and disparities in the standard of obstetric and neonatal care between the study settings. To that end, the current study included referred mothers from local health centres for primary CDs only, which increases the risk of fetomaternal complications.

Our study also found that peripartum hysterectomy, the need for ICU admission and urethral/bowel injury were higher among mothers who underwent CD at the second stage of labour; however, the small number of those complications are insufficient to conclude.

On the other hand, we observed a significantly higher association between CD performed at the second stage of labour and adverse neonatal outcomes, including a fifth min Apgar score <7, the need for NICU admission and neonatal death than CD performed at the first stage of labour.

In agreement with our findings, previous studies^13 22^ showed a significant association between the second stage of CD and neonatal morbidity, including an increased incidence of fifth min Apgar score <7 and higher NICU admission rates, but failed to show an association with neonatal death. In disagreement, our findings show that the CD performed at the second stage of labour is not only associated with neonatal morbidity but also with neonatal mortality, in line with other studies.^14 22 26^

On the contrary, some studies even observed the insignificant impact of CD performed at the second stage of labour on adverse neonatal outcomes.^15 17 23–25^ These might indicate that the mode of delivery being emergency caesarean section, referred from other centres and the poor quality of neonatal ICUs in our study settings could justify the observed difference, suggesting the need for improving intensive neonatal care in resource-limited settings. Also, obstetric surgical access is limited in African countries and is responsible for increasing referrals to tertiary centres; when performed, it is often too late to reduce perinatal deaths and is associated with poorer neonatal outcomes, especially during emergency CD.^8^

Thus, providing equitable obstetric surgical access and strengthening obstetric surgical and intensive neonatal care in low-income countries need immediate attention to improve maternal and perinatal outcomes.^26–29^

### Strength and limitations

The strength of our study is that only referred mothers are included as a source population, which allows a comparison of the obstetric outcomes of these underserved populations to exemplify the existing problems. However, the retrospective nature of the study design precludes the inclusion of the most significant covariates, which might affect the impact of the stage of labour on the outcome variables. We also relied on data retrieved from the medical records of a single institution, making it difficult to extrapolate the findings to the non-referred mothers in the study setting. Furthermore, because of substandard overall NICU care and poor documentation in our hospital, we failed to include additional adverse neonatal outcomes, such as septicaemia, intubation, seizures and fetal injuries. Therefore, a further prospective study, including large sample size, multiple study hospitals and various neonatal outcomes, is needed to determine the generalisability of the findings to the entire study population. Despite these limitations, obstetric care providers should consider the impact of second-stage CDs and provide effective management to lower the burden of such cases.

## Conclusion

CS performed at the second stage of labour among referred mothers is associated with an increased risk of adverse maternal and neonatal outcomes. Thus, strengthening and improving obstetric emergency surgical services and intensive neonatal care for those populations would help reduce poor maternal and fetal outcomes. Further investigation may also require improving obstetric and neonatal intensive care.

## Supplementary Material

Reviewer comments

Author's manuscript

## Data Availability

Data are available on reasonable request.

## References

[1] Betrán AP, Ye J, Moller A-B, et al. The increasing trend in caesarean section rates: global, regional and national estimates: 1990-2014. PLoS One 2016;11:e0148343. 10.1371/journal.pone.0148343 26849801 PMC4743929

[2] Gedefaw G, Demis A, Alemnew B, et al. Prevalence, indications, and outcomes of caesarean section deliveries in Ethiopia: a systematic review and meta-analysis. Patient Saf Surg 2020;14:11. 10.1186/s13037-020-00236-8 32292491 PMC7140488

[3] Elias S, Wolde Z, Tantu T, et al. Determinants of early neonatal outcomes after emergency cesarean delivery at Hawassa University comprehensive specialised hospital, Hawassa, Ethiopia. PLoS One 2022;17:e0263837. 10.1371/journal.pone.0263837 35324921 PMC8947390

[4] Zewdu D, Tantu T. Incidence and predictors of severe postpartum hemorrhage after cesarean delivery in South central Ethiopia: a retrospective cohort study. Sci Rep 2023;13:3635. 10.1038/s41598-023-30839-x 36869166 PMC9984450

[5] Sobhy S, Arroyo-Manzano D, Murugesu N, et al. Maternal and perinatal mortality and complications associated with caesarean section in low-income and middle-income countries: a systematic review and meta-analysis. The Lancet 2019;393:1973–82. 10.1016/S0140-6736(18)32386-9 30929893

[6] Ebener S, Stenberg K, Brun M, et al. Proposing standardised geographical indicators of physical access to emergency obstetric and newborn care in low-income and middle-income countries. BMJ Glob Health 2019;4(Suppl 5):e000778. 10.1136/bmjgh-2018-000778 PMC6623986 31354979

[7] Irani M, Deering S. Challenges affecting access to cesarean delivery and strategies to overcome them in low-income countries. Int J Gynaecol Obstet 2015;131:30–4. 10.1016/j.ijgo.2015.04.036 26115791

[8] Shah A, Fawole B, M’imunya JM, et al. Cesarean delivery outcomes from the WHO global survey on maternal and perinatal health in Africa. Int J Gynaecol Obstet 2009;107:191–7. 10.1016/j.ijgo.2009.08.013 19782977

[9] Dalvi SA. Difficult deliveries in cesarean section. J Obstet Gynaecol India 2018;68:344–8. 10.1007/s13224-017-1052-x 30224836 PMC6133784

[10] Lipschuetz M, Guedalia J, Cohen SM, et al. Unplanned cesarean delivery in the second stage of labor holds higher odds of complications than in the first stage, while similar in primiparas and multiparas. Fetal Diagn Ther 2020;47:565–71. 10.1159/000504573 31982884

[11] Manning JB, Tolcher MC, Chandraharan E, et al. Delivery of an impacted fetal head during caesarian—A literature review and proposed management algorithm. Obstet Gynecol Surv 2015;70:719–24. 10.1097/OGX.0000000000000248 26584721

[12] Asıcıoglu O, Güngördük K, Yildirim G, et al. Second-stage vs first-stage caesarean delivery: comparison of maternal and perinatal outcomes. J Obstet Gynaecol 2014;34:598–604. 10.3109/01443615.2014.920790 24911878

[13] Sinha S, Malik S, Dixit M. First versus second stage caesarean section: a comparison of maternal and neonatal outcomes. Int J Reprod Contracept Obstet Gynecol 2017;6:3477. 10.18203/2320-1770.ijrcog20173467

[14] Seal SL, Kamilya G, Mukherji J, et al. Outcome in second-versus first-stage cesarean delivery in a teaching institution in Eastern India. Am J Perinatol 2010;27:507–12. 10.1055/s-0030-1248936 20175041

[15] Sung JF, Daniels KI, Brodzinsky L, et al. Cesarean delivery outcomes after a prolonged second stage of labor. Am J Obstet Gynecol 2007;197:306. 10.1016/j.ajog.2007.07.005 17826431

[16] Wilkof-Segev R, Naeh A, Barda S, et al. Unintended uterine extension at the time of cesarean delivery – risk factors and associated adverse maternal and neonatal outcomes. J Matern-Fetal Neonatal Med 2023;36:2204997. 10.1080/14767058.2023.2204997 37127602

[17] Alexander JM, Leveno KJ, Rouse DJ, et al. Comparison of maternal and infant outcomes from primary cesarean delivery during the second compared with first stage of labor. Obstet Gynecol 2007;109:917–21. 10.1097/01.AOG.0000257121.56126.fe 17400854

[18] Sucak A, Celen S, Akbaba E, et al. Comparison of nulliparas undergoing cesarean section in first and second stages of labour: a prospective study in a tertiary teaching hospital. Obstet Gynecol Int 2011;2011:986506. 10.1155/2011/986506 21941557 PMC3176624

[19] Lurie S, Raz N, Boaz M, et al. Comparison of maternal outcomes from primary cesarean section during the second compared with first stage of labor by indication for the operation. Eur J Obstet Gynecol Reprod Biol 2014;182:43–7. 10.1016/j.ejogrb.2014.08.025 25218551

[20] Vitner D, Bleicher I, Levy E, et al. Differences in outcomes between cesarean section in the second versus the first stages of labor. J Matern Fetal Neonatal Med 2019;32:2539–42. 10.1080/14767058.2018.1440545 29471705

[21] Cebekulu L, Buchmann EJ. Complications associated with cesarean section in the second stage of labor. Int J Gynaecol Obstet 2006;95:110–4. 10.1016/j.ijgo.2006.06.026 16934268

[22] Pergialiotis V, Vlachos DG, Rodolakis A, et al. First versus second stage C/S maternal and neonatal morbidity: a systematic review and meta-analysis. Eur J Obstet Gynecol Reprod Biol 2014;175:15–24. 10.1016/j.ejogrb.2013.12.033 24447469

[23] Thuillier C, Roy S, Peyronnet V, et al. Impact of recommended changes in labor management for prevention of the primary cesarean delivery. Am J Obstet Gynecol 2018;218:341. 10.1016/j.ajog.2017.12.228 29291413

[24] Belay T, Yusuf L, Negash S. A comparative study on first stage versus second stage caesarean section on maternal and perinatal outcome. Ethiop Med J 2014;52:1–8. 25069208

[25] Radha P, Tagore S, Rahman MFA, et al. Maternal and perinatal morbidity after caesarean delivery at full cervical dilatation. Singapore Med J 2012;53:655–8. 23112016

[26] Zewdu D, Tantu T, Degemu F, et al. Exploring factors influencing skin Incision to the delivery time and their impact on neonatal outcomes among emergency cesarean deliveries indicated for non-reassured fetal heart rate status. Front Pediatr 2023;11:1224508. 10.3389/fped.2023.1224508 37808561 PMC10551621

[27] Ochieng Arunda M, Agardh A, Asamoah BO. Cesarean delivery and associated socioeconomic factors and neonatal survival outcome in Kenya and Tanzania: analysis of national survey data. Glob Health Action 2020;13:1748403. 10.1080/16549716.2020.1748403 32345146 PMC7241493

[28] Epiu I, Byamugisha J, Kwikiriza A, et al. Health and sustainable development; strengthening peri-operative care in low-income countries to improve maternal and neonatal outcomes. Reprod Health 2018;15:168. 10.1186/s12978-018-0604-6 30290812 PMC6173895

[29] Zewdu D, Tantu T, Ali R, et al. Prevalence and predictors of postpartum anemia after caesarean delivery in Ethiopia: a retrospective analysis of risk factors. Midwifery 2023;123. 10.1016/j.midw.2023.103707 37187101

