# Supplementary material for: Association between the stage of labour during caesarean delivery with adverse maternal and neonatal outcomes among referred mothers to tertiary centres in resource-limited settings
Source: BMJ Open. 2023 Nov 28;13(11):e077265. doi: 10.1136/bmjopen-2023-077265 (PMC10685916; doi:10.1136/bmjopen-2023-077265)
Supplement: Author's manuscript [file bmjopen-2023-077265.draft_revisions.pdf]

BMJ Open

BMJ Open is committed to open peer review. As part of this commitment we make the peer review history of every article we publish publicly available.

When an article is published we post the peer reviewers' comments and the authors' responses online. We also post the versions of the paper that were used during peer review. These are the versions that the peer review comments apply to.

The versions of the paper that follow are the versions that were submitted during the peer review process. They are not the versions of record or the final published versions. They should not be cited or distributed as the published version of this manuscript.

BMJ Open is an open access journal and the full, final, typeset and author-corrected version of record of the manuscript is available on our site with no access controls, subscription charges or pay-per-view fees (http://bmjopen.bmj.com).

If you have any questions on BMJ Open's open peer review process please email info.bmjopen@bmj.com

BMJ Open

Association between the stage of labor during cesarean delivery with adverse maternal and neonatal outcomes among referred mothers to tertiary centers in resource-limited settings

Journal:	*BMJ Open*
Manuscript ID	bmjopen-2023-077265
Article Type:	Original research
Date Submitted by the Author:	29-Jun-2023
Complete List of Authors:	Zewdu, Dereje ; Wolkite University, Department of Anesthesia<br>Tantu, Temesgen ; Wolkite University, Obstetrics and Gynecology<br>Degemu, Fikretsion; Wolkite University, Department of Pediatrics and Child Health<br>Abdlwehab, Mukerem; Wolkite University, Department of Pediatrics and Child Health
Keywords:	OBSTETRICS, Anaesthesia in obstetrics < ANAESTHETICS, NEONATOLOGY

SCHOLARONE™
Manuscripts

I, the Submitting Author has the right to grant and does grant on behalf of all authors of the Work (as defined in the below author licence), an exclusive licence and/or a non-exclusive licence for contributions from authors who are: i) UK Crown employees; ii) where BMJ has agreed a CC-BY licence shall apply, and/or iii) in accordance with the terms applicable for US Federal Government officers or employees acting as part of their official duties; on a worldwide, perpetual, irrevocable, royalty-free basis to BMJ Publishing Group Ltd ("BMJ") its licensees and where the relevant Journal is co-owned by BMJ to the co-owners of the Journal, to publish the Work in this journal and any other BMJ products and to exploit all rights, as set out in our licence.

The Submitting Author accepts and understands that any supply made under these terms is made by BMJ to the Submitting Author unless you are acting as an employee on behalf of your employer or a postgraduate student of an affiliated institution which is paying any applicable article publishing charge ("APC") for Open Access articles. Where the Submitting Author wishes to make the Work available on an Open Access basis (and intends to pay the relevant APC), the terms of reuse of such Open Access shall be governed by a Creative Commons licence – details of these licences and which Creative Commons licence will apply to this Work are set out in our licence referred to above.

Other than as permitted in any relevant BMJ Author's Self Archiving Policies, I confirm this Work has not been accepted for publication elsewhere, is not being considered for publication elsewhere and does not duplicate material already published. I confirm all authors consent to publication of this Work and authorise the granting of this licence.

For peer review only

Association between the stage of labor during cesarean delivery with adverse maternal and neonatal outcomes among referred mothers to tertiary centers in resource-limited settings

Dereje Zewdu[1]*, Temesgen Tantu[2], Fikretsion Degemu[3], Mukerem Abdlwehab[3]

[1]Department of Anesthesia, College of Medicine and Health Science, Wolkite University, Wolkite, Ethiopia

[2]Department of Obstetrics and Gynecology, College of Medicine and Health Science, Wolkite University, Wolkite, Ethiopia

[3]Department of Pediatrics and Child Health, College of Medicine and Health Science, Wolkite University, Wolkite, Ethiopia

*Corresponding author

Name: Dereje Zewdu

Email: Derejezewdu1529@gmail.com

Phone Number: +251 9240 4631

For peer review only

Abstract

Objective: Although the cesarean deliveries (CD) rate has substantially increased, little is known about the impacts of CD performed in the first and second stages of labor on fetomaternal outcomes, especially among referred mothers. The study aimed to investigate the association between cesarean deliveries (CD) performed in the first and second stages of labor and poor maternal and neonatal outcomes among referred mothers to tertiary centers.

Setting: A retrospective cohort study was conducted using medical records of mother-infant pairs from September 2020 to May 2023in Southern Ethiopia.

Participants: We retrospectively collected data from 848 participants who underwent emergency CD on a referral basis during the study period.

Primary outcome measure: The primary outcomes of interest were adverse maternal and neonatal outcomes. Data were analyzed using descriptive and inferential statistics.

Results: Of the 848 Cesarean deliveries, 722 (85.2%) and 126 (14.8%) were performed at the first and second stages of labor, respectively. Cesarean deliveries performed at the second stage were associated with nulliparity, increased maternal age, and birth weight. Compared with the first-stage CD, the second-stage CD was associated with a significantly increased risk of adverse maternal (OR = 3.7, 95% CI 2.4–5.7) and neonatal outcomes (OR 2.0; 95% CI 1.3–2.9), including neonatal death.

Conclusion: Second-stage CDs have an increased risk of adverse maternal and neonatal outcomes. Strengthening the referral system with local health centers and improving obstetric emergency surgical services and intensive neonatal care for those populations would help to decrease the maternal and fetal consequences.

Keywords: Cesarean delivery, Stage of Labor, maternal adverse outcomes, neonatal adverse outcomes

For peer review only - http://bmjopen.bmj.com/site/about/guidelines.xhtml

Article summary

Strengths and limitations of this study

- The study evaluated the impact of second-stage CD on fetomaternal outcomes among referred mothers and that was not studied so far.
- The findings provide insight for clinicians to develop guidelines to minimize the burden of those populations.
- The study included 3-year data, which is relatively representative of the existing problems.
- The retrospective nature of the study design may result in variable selection bias.
- Extrapolating the findings to non-referred participants might be difficult.

For peer review only

For peer review only - http://bmjopen.bmj.com/site/about/guidelines.xhtml

Introduction

The cesarean delivery (CD) rate has significantly increased globally, representing one-third of total institutional deliveries in Ethiopia.[1, 2] Although CD is popular and usually lifesaving, it may increase maternal and neonatal adverse outcomes and health care expenses.[3, 4]

In many low-income countries where equitable access to obstetric surgical services is limited, the patient frequently gets referred to nearby tertiary centers, which tends to be associated with compromised obstetric outcomes.[5, 6] Increased access to cesarean delivery in resource-limited settings could help reduce the incidence of maternal and newborn morbidity and mortality.[7] Nonetheless, being referred to other centers for CD and delays in timely intervention contribute to the likelihood of unfavorable fetomaternal outcomes.[8] Despite these, the impact of the stage of labor during CD on maternal and neonatal adverse outcomes among those populations has not been well-established. While clinicians carried out many CD procedures during the first stage of labor with fewer undesirable outcomes for the mother or the newborn, the second-stage CD is technically more difficult and poses serious consequences.[9, 10]

Previous studies demonstrated inconclusive and conflicting results on the association between CD at the stage of labor and fetomaternal outcomes. Some studies have found a significant correlation between CD performed at the second stage of labor and adverse maternal outcomes.[11-18] On the contrary, others found no association between second-stage CD and poor maternal outcomes.[19-21] Poor maternal outcomes among CD performed at the second stage of labor include increased blood loss, uterine atony, need for blood transfusion, and perioperative complications.

Concerning neonatal outcomes, one study reported serious neonatal complications are common in the CD performed at the first stage of labor.[10] Nevertheless, other studies found higher poor outcomes in second-stage CD [13, 14, 21, 22] and no association between these measurements. [15, 17, 23-25] Neonatal adverse outcomes include a low 5th-minute Apgar score, increased need for NICU admission, and neonatal death.

As far as our search, no other studies have compared the association between CD performed at the first and second stage of labor on their neonatal and maternal outcomes among referred mothers to tertiary centers. For practitioners to pinpoint the existing clinical gap and utilize the

For peer review only - http://bmjopen.bmj.com/site/about/guidelines.xhtml

findings to improve clinical practice, it would be beneficial to understand the connections between CD carried out at the first and second stages of labor and undesirable newborn and mother outcomes.

Our aim, therefore, was to investigate the association between CS performed at the first and second stage of labor with adverse maternal and neonatal outcomes among mothers referred to Wolkite University Referral and Teaching Hospital in Southern Ethiopia. The findings of this study provide information for clinicians, researchers, and policymakers to maximize the quality of perioperative obstetric and intensive neonatal care and decrease second-stage CD-related fetomaternal complications.

Methods

Study population and study design

This retrospective cohort study included all consecutive women who underwent primary cesarean section for singleton term pregnancy between September 2020 and May 2023 at Wolkite University Specialized and Teaching Hospital in Ethiopia.

We have conducted the study per the Declaration of Helsinki Ethical Principles for Medical Research involving human subjects protocol. The Wolkite University Ethical Review Board provided the ethical clearance for the study; informed written consent was exempted from data collection because of the retrospective nature of the study design.

A total of 848 consecutive primary cesarean deliveries performed for referred mothers during the study period were included for final analysis. Exclusion criteria were as follows: mothers with previous cesarean delivery, multiple gestation, preterm birth, intrauterine growth restriction, fetal anomalies, and incomplete documentation.

Data collection

We retrospectively collected data from the maternal and neonatal medical records, including maternal and neonatal baseline characteristics, perioperative data, and adverse maternal and neonatal outcomes of all study participants using a checklist adapted from prior studies.

For peer review only - http://bmjopen.bmj.com/site/about/guidelines.xhtml

The maternal and neonatal baseline characteristics included maternal age, body mass index, parity, residency area, educational status, onset and duration of labor, gestational age, birth weight, and fetal sex. Perioperative maternal and neonatal characteristics included skin incision to delivery time, duration of operation, estimated blood loss, uterine atony, unintentional uterine extension, uterine artery ligation, need for blood transfusion, Ureteral/bowel injury, perioperative hysterectomy, need for ICU admission, and maternal death, as well as 5th minute Apgar score, need for NICU admission, and neonatal death.

Study outcomes

The primary outcomes of interest were adverse maternal and neonatal outcomes. The adverse maternal outcomes included one or more of the following: perioperative hemorrhage, uterine artery ligation, unintentional uterine extension, ureteral or bowel injury, peripartum hysterectomy, and maternal death. Neonatal adverse outcomes included one or more of the following: 5th minute Apgar score < 7, need for NICU admission, and neonatal death.

Statistical analysis

We entered, coded, and analyzed the data using SPSS (Statistical Package for the Social Sciences for Windows version 26.0, SPSS Inc., and Chicago, Illinois, USA). We used the student t-test test for numerical variables and described them as mean ± SD.

We presented the categorical data as frequency and percentage, and the association between independent and dependent variables was analyzed using the Chi-square and Fisher exact tests for statistical differences between groups. Logistic regression analysis was used to examine the impacts of CS performed at the first and second stages of labor on maternal and neonatal adverse outcomes. The results were displayed as an odd ratio with 95% CI (confidence interval). A p-value < 0.05 was considered statistically significant.

Patient and public involvement

We did not involve patients and public in the study—we simply retrieved data from the medical records.

Results

For peer review only

For peer review only - http://bmjopen.bmj.com/site/about/guidelines.xhtml

Maternal and neonatal baseline characteristics

Of 897 women who underwent primary cesarean section performed during the study period, about 848 (94.5%) were included for final analysis. Of these, 722 (85.1%) cesarean deliveries were performed in the first stage and 126 ((14.9%) in the second stage of labor. While education, referral status, the labor onset, and fetal sex between CS performed at the first and second stages were comparable, nulliparity, the onset of labor to delivery time, and birthweight were significantly higher in the second-stage CD [58.7% vs. 35.7%; p-value < 0.001], [14.8 ± 10.4 vs. 10.6 ± 7.1; p-value < 0.001], and [3298.1 ± 338.3 vs. 3444.1 ± 322.3; p-value < 0.001], respectively as shown (Table 1).

Table 1: Maternal and neonatal baseline characteristics between CS performed at the first and second stages of labor

Variables	CS at 1st stage of labor (n=722)	CS at 2nd stage of labor (n=126)	p-value
Maternal age, year, mean ± SD	26.5 ± 4.8	24.9 ± 4.5	**< 0.001**
BMI, Kg/m^2, mean ± SD	27.9 ± 2.5	28.6 ± 2.5	0.004
Nulliparity, n (%)			**< 0.001**
Yes	258 (35.7)	74 (58.7)	
No	464 (64.3)	52 (41.3)	
Educational status, n (%)			0.17
Literate	588 (81.4)	96 (76.2)	
Illiterate	134 (18.6)	30 (23.8)	
Referral status, n (%)			0.62
Rural	338 (46.8)	62 (49.2)	
Urban	384 (53.2)	64 (50.8)	
The onset of labor, n (%)			0.65
Spontaneous	662 (91.7)	114 (90.5)	
Induced	60 (8.3)	12 (9.5)	
Labor onset to delivery, hours, mean ± SD	10.6 ± 7.1	14.8 ± 10.4	**< 0.001**
GA at delivery, weeks, mean ± SD	38.6 ± 1.3	38.7 ± 1.5	0.54

For peer review only - http://bmjopen.bmj.com/site/about/guidelines.xhtml

Birthweight at delivery, gram, mean ± SD	3298.1 ± 338.3	3444.1 ± 322.3	**< 0.001**
Fetal sex, n (%)			0.9
Male	375 (51.9)	66 52.4)	
Female	347 (48.1)	60 (47.6)	

BMI: body mass index, SD: standard deviation, P < 0.05 is considered significant.

Perioperative maternal and neonatal characteristics

maternal perioperative characteristics, as shown in (Table 2), the CS performed at the second stage of labor was significantly associated with prolonged skin incision-to-delivery time, operation time, postpartum hemorrhage, uterine atony, uterine artery ligation, need for blood transfusion, unintentional uterine artery extension, perioperative hysterectomy, need for ICU admission, and even ureteral/bowel injury (p < 0.001, p < < 0.001, p < 0.001, p < 0.001, p = 0.002, p = 0.008, p < 0.001, p = 0.016, p = 0.026, p = 0.036, respectively).

Similarly, neonatal outcomes, including 5th minute Apgar score < 7, need for NICU admission, and neonatal death were significantly high in CS performed at the second stage than in the first stage of labor (22.2% vs. 8.3%; p < 0.001, 34.9% vs. 24.6%; p < 0.001, 14.3% vs. 3.6%; p < 0.001), respectively as shown in (Table 2).

Table 2: Perioperative maternal and neonatal characteristics between CS performed at the first and second stages of labor

Variables	CS at the first stage of labor (n=722)	CS at the second stage of labor (n=126)	p-value
Skin incision to delivery time, minutes	6.1 ± 1.6	7.7 ± 2.2	**< 0.001**
Duration of operation, minutes	41.8 ± 20.1	49.4 ± 26.1	**< 0.001**
Duration of operation >1 hour, n (%)			**< 0.001**
Yes	122 (16.9)	42 (33.4)	
No	600 (83.1)	84 (66.6)	
Estimated blood loss, ml	464.5 ± 342.2	599.2 ± 539.2	**< 0.001**
Perioperative hemorrhage (> 1000ml), n (%)			**< 0.001**

For peer review only - http://bmjopen.bmj.com/site/about/guidelines.xhtml

Yes	44 (6.1)	20 (15.9)	
No	678 (93.9)	106 (84.1)	
Uterine atony, n (%)			**< 0.001**
Yes	34 (4.7)	18 (14.3)	
No	688 (95.3)	108 (85.7)	
Uterine artery ligation, n (%)	18 (2.5)	10 (7.9)	0.002
Blood transfusion, n (%)			0.008
Yes	22 (3)	10 (7.9)	
No	700 (97)	116 (92.1)	
Unintentional uterine extension, n (%)	4 (0.5)	7 (5.7)	**< 0.001**
Perioperative hysterectomy, n (%)	8 (1.1)	5 (4.1)	0.016
ICU admission, n (%)	12 (1.6)	6 (4.9)	0.026
Ureteral/bowel injury, n (%)	4 (0.5)	3 (2.4)	0.036[a]
Maternal death, n (%)	3 (0.4)	2 (1.6)	0.08
**Neonatal**			
5th minute Apgar score < 7, n (%)			**< 0.001**
Yes	60 (8.3)	28 (22.2)	
No	662 (91.7)	98 (77.8)	
Need for NICU admission, n (%)			**< 0.001**
Yes	178 (24.6)	44 (34.9)	
No	544 (75.4)	82 (65.1)	
Neonatal death, n (%)			**< 0.001**
Yes	26 (3.6)	18 (14.3)	
No	696 (96.4)	108 (85.7)	

NICU: neonatal intensive care unit, ICU: intensive care unit, CS: cesarean section, [a:] Fisher exacts test

Association between CD performed at the first and second stage of labor with adverse maternal and neonatal outcomes

For peer review only - http://bmjopen.bmj.com/site/about/guidelines.xhtml

Logistic regression analysis showed that the likelihood of developing adverse maternal outcomes after CS performed at the second stage of labor was 3.7 times (OR = 3.7, 95% CI 2.4 – 5.7) higher than the first-stage CD. Similarly, second-stage CD increases the risk of developing adverse neonatal outcomes 2-fold (OR 2.0; 95% CI 1.3 – 2.9) compared to CS performed at the first stage of labor, as shown in (Table 3)

.

Table 3: Association of adverse maternal and neonatal outcomes with CS performed at the first and second stages of labor

Outcomes	CS at the first stage of labor (n=722)	CS at the second stage of labor (n=126)	Odd ratios (95% CI)	p-value
Adverse maternal outcomes				**<0.001**
Yes	88 (12.2)	43 (34.1)	3.7 (2.4 – 5.7)	
No	634 (87.8)	88 (65.9)	1	
Adverse neonatal outcomes				**<0.001**
Yes	198 (27.4)	54 (42.8)	2.0 (1.3 – 2.9)	
No	524 (72.6)	72 (57.2)	1	

CS: cesarean section, CI: confidence interval

Discussion

In this retrospective study involving 848 women who underwent emergency cesarean delivery at a referral hospital in Southern Ethiopia, we compared maternal and neonatal adverse outcomes of CS performed during the first and second stages of labor. Our main findings were that CS performed at the second stage was associated with an increased risk of maternal and neonatal adverse outcomes compared to CS performed at the first stage of labor.

For peer review only - http://bmjopen.bmj.com/site/about/guidelines.xhtml

Increased adverse maternal outcomes included longer operation times, estimated blood loss or postpartum hemorrhage, uterine atony, and uterine artery ligation procedures, suggesting that CS at the second stage might be technically challenging and associated with higher odds of complications.[9, 10] This is likely due to the engagement of the fetal head in the pelvis, the difficulty in delineating the bladder, and the larger infants that are perhaps more difficult to remove from the uterus.[11, 12]

The increased amount of blood loss because of uterine atony and unintentional uterine extensions is frequent in the second stage CD, as revealed in the present and previous studies.[13, 14] Other studies also showed that unintended uterine extension was associated with longer operation time, more blood loss, postpartum anemia, and an increased need for blood transfusion.[15, 16]

We believe that the extended labor duration and more substantial uterine fatigue may be the reason for the increase in uterine atony incidence in the second stage CD.[17] Our study also showed because of the increased amount of blood loss and uterine atony, an increased need for blood transfusion and uterine artery ligation procedures are more frequent in second-stage CD, in line with previous reports.[18] Therefore, it is crucial to consider effective uterotonic agents, leveraging the expertise of experienced obstetricians, and continual monitoring to lessen maternal morbidity in these scenarios.

Contradicting our findings, a few studies [19-21] reported no association between second-stage CD and more blood loss, uterine atony, and other poor maternal outcomes. These discrepancies may occur because of the variations in the study population recruited for analysis (urgency of the surgery, indications for CD, and delayed presentation in their referral status), fluctuations in sample size and study design, and disparities in the standard of obstetric and neonatal care between the study settings. To that end, the current study included referred mothers from local health centers for primary CDs only, which increases the risk of fetomaternal complications.

Our study also found that peripartum hysterectomy, the need for ICU admission, and urethral/bowel injury were higher among mothers who underwent CS at the second stage of labor; however, the small number of those complications are insufficient to conclude.

For peer review only - http://bmjopen.bmj.com/site/about/guidelines.xhtml

On the other hand, we observed a significantly higher association between CS performed at the second stage of labor and adverse neonatal outcomes, including a 5th-minute Apgar score < 7, the need for NICU admission, and neonatal death than CS performed at the first stage of labor.

In agreement with our findings, previous studies [13, and 22] showed a significant association between the second stage of CD and neonatal morbidity, including an increased incidence of 5th minute Apgar score < 7 and higher NICU admission rates, but failed to show an association with neonatal death. In disagreement, our findings show that the CS performed at the second stage of labor is not only associated with neonatal morbidity but also with neonatal mortality, in line with other studies.[14, 22]

On the contrary, some studies even observed the insignificant impact of CD performed at the second stage of labor on adverse neonatal outcomes.[15, 17, 23-25] These might indicate that the mode of delivery being emergency cesarean section, referred from other centers, and the poor quality of neonatal intensive care units in our study settings could justify the observed difference, suggesting the need for improving intensive neonatal care in resource-limited settings. Also, obstetric surgical access is limited in African countries and is responsible for increasing referrals to tertiary centers; when performed, it is often too late to reduce perinatal deaths and is associated with poorer neonatal outcomes, especially during emergency CS.[8]

Thus, providing equitable obstetric surgical access and strengthening obstetric surgical and intensive neonatal care in low-income countries need immediate attention to improve maternal and perinatal outcomes.[26, 27]

Strength and limitations

The strength of our study is that only referred mothers are included as a source population, which allows a comparison of the obstetric outcomes of these underserved populations to exemplify the existing problems. However, the retrospective nature of the study design precludes the inclusion of the most significant covariates, which might affect the impact of the stage of labor on the outcome variables. We also relied on data retrieved from the medical records of a single institution, making it difficult to extrapolate the findings to the non-referred mothers in the study setting. Therefore, a further prospective study, including large sample size and multiple study hospitals, is needed to determine the generalizability of the findings to the entire study population. Despite these limitations, obstetric care providers should consider the impact of

For peer review only - http://bmjopen.bmj.com/site/about/guidelines.xhtml

second-stage cesarean deliveries and provide effective management to lower the burden of such cases.

Conclusion

CS performed at the second stage of labor among referred mothers is associated with an increased risk of adverse maternal and neonatal outcomes. Thus, strengthening the referral system with catchment health centers and improving obstetric emergency surgical services and intensive neonatal care for those populations would help to decrease the maternal and fetal consequences. Further investigation on how to improve the referral system, obstetric and neonatal intensive care may need.

Acknowledgments

The authors acknowledge Wolkite University's Specialized and Teaching Hospital Management, data collectors, and medical record office staff for their invaluable support.

Contributors

DZ, TT, FD, and MA conceived and designed the study. DZ and TT performed the data collection, data analysis, and initial draft manuscript. DZ and MA reviewed the manuscript critically. All authors read and approved the final manuscript.

Funding

The authors have not declared a specific grant for this research from any funding agency in the public, commercial, or not-for-profit sectors.

Competing interests

For peer review only - http://bmjopen.bmj.com/site/about/guidelines.xhtml

None declared.

Patient consent

Not required.

Ethics approval

Wolkite University Ethical Review Board (ERB) provided the ethical clearance for the study, and written consent was exempted by ERB because of the retrospective nature of the study design.

Provenance and peer review

Not commissioned; externally peer-reviewed.

Data sharing statement

All data supporting our findings will be shared on request.

Contact

Derejezewdu1529@gmail.com

For peer review only

For peer review only - http://bmjopen.bmj.com/site/about/guidelines.xhtml

References

1. Betrán AP, Ye J, Moller AB, Zhang J, Gülmezoglu AM, Torloni MR. The increasing trend in cesarean section rates: global, regional and national estimates: 1990-2014. PloS one. 2016 Feb 5; 11(2):e0148343.
2. Gedefaw G, Demis A, Alemnew B, Wondmieneh A, Getie A, Waltengus F. Prevalence, indications, and outcomes of caesarean section deliveries in Ethiopia: a systematic review and meta-analysis. Patient safety in surgery. 2020 Dec; 14(1):1-0.
3. Elias S, Wolde Z, Tantu T, Gunta M, Zewudu D. Determinants of early neonatal outcomes after emergency cesarean delivery at Hawassa University comprehensive specialised hospital, Hawassa, Ethiopia. Plos one. 2022 Mar 24; 17(3):e0263837.
4. Zewdu D, Tantu T. Incidence and predictors of severe postpartum hemorrhage after cesarean delivery in South Central Ethiopia: a retrospective cohort study. Scientific Reports. 2023 Mar 3; 13(1):3635.
5. Sobhy S, Arroyo-Manzano D, Murugesu N, Karthikeyan G, Kumar V, Kaur I, Fernandez E, Gundabattula SR, Betran AP, Khan K, Zamora J. Maternal and perinatal mortality and complications associated with caesarean section in low-income and middle-income countries: a systematic review and meta-analysis. The Lancet. 2019 May 11; 393(10184):1973-82.
6. Ebener S, Stenberg K, Brun M, Monet JP, Ray N, Sobel HL, Roos N, Gault P, Conlon CM, Bailey P, Moran AC. Proposing standardised geographical indicators of physical access to emergency obstetric and newborn care in low-income and middle-income countries. BMJ global health. 2019 Jun 1; 4(Suppl 5):e000778.

For peer review only - http://bmjopen.bmj.com/site/about/guidelines.xhtml

7. Irani M, Deering S. Challenges affecting access to cesarean delivery and strategies to overcome them in low-income countries. International Journal of Gynecology & Obstetrics. 2015 Oct 1; 131(1):30-4.
8. Shah A, Fawole B, M'imunya JM, Amokrane F, Nafiou I, Wolomby JJ, Mugerwa K, Neves I, Nguti R, Kublickas M, Mathai M. Cesarean delivery outcomes from the WHO global survey on maternal and perinatal health in Africa. International Journal of Gynecology & Obstetrics. 2009 Dec 1; 107(3):191-7.
9. Dalvi SA. Difficult deliveries in cesarean section. the Journal of Obstetrics and Gynecology of India. 2018 Oct; 68(5):344-8.
10. Lipschuetz M, Guedalia J, Cohen SM, Lewkowicz AA, Amsalem H, Karavani G, Yagel S, Unger R. Unplanned cesarean delivery in the second stage of labor holds higher odds of complications than in the first stage, while similar in primiparas and multiparas. Fetal Diagnosis and Therapy. 2020; 47(7):565-71.
11. Jennifer B Manning, Mary C Tolecher, Edwin C, et al: Delivery of an Impacted fetal head during Caesarian—A literature review and proposed management algorithm. Obstet Gynecol Surv. 2015; 70(11):719–24.
12. Asıcıoglu O, Güngördük K, Yildirim G, Asıcıoglu BB, Güngördük ÖÇ, Ark C, Günay T, Yenigül N. Second-stage vs first-stage caesarean delivery: comparison of maternal and perinatal outcomes. Journal of Obstetrics and Gynaecology. 2014 Oct 1; 34(7):598-604.
13. Sinha S, Malik S, Dixit M. First versus second stage caesarean section: a comparison of maternal and neonatal outcomes. International Journal of Reproduction, Contraception, Obstetrics and Gynecology. 2017 Aug 1; 6(8):3477-81.
14. Seal SL, Kamilya G, Mukherji J, Bhattacharyya SK, De A, Hazra A. Outcome in second- versus first-stage cesarean delivery in a teaching institution in eastern India. American Journal of Perinatology. 2010 Jun; 27(06):507-12.
15. Sung JF, Daniels KI, Brodzinsky L, El-Sayed YY, Caughey AB, Lyell DJ. Cesarean delivery outcomes after a prolonged second stage of labor. American Journal of Obstetrics and Gynecology. 2007 Sep 1; 197(3):306-e1.
16. Renana Wilkof-Segev, Amir Naeh, Sivan Barda, Mordechai Hallak & Rinat Gabbay-Benziv (2023) Unintended uterine extension at the time of cesarean delivery – risk

For peer review only - http://bmjopen.bmj.com/site/about/guidelines.xhtml

factors and associated adverse maternal and neonatal outcomes, The Journal of Maternal-Fetal & Neonatal Medicine, 36:1, 2204997, DOI: 10.1080/14767058.2023.2204997

17. Alexander JM, Leveno KJ, Rouse DJ, Landon MB, Gilbert S, Spong CY, Varner MW, Moawad AH, Caritis SN, Harper M, Wapner RJ. Comparison of maternal and infant outcomes from primary cesarean delivery during the second compared with first stage of labor. Obstetrics & Gynecology. 2007 Apr 1; 109(4):917-21.
18. Sucak A, Çelen Ş, Akbaba E, Soysal S, Moraloglu O, Danışman N. Comparison of nulliparas undergoing cesarean section in first and second stages of labour: a prospective study in a tertiary teaching hospital. Obstetrics and gynecology international. 2011 Jan 1; 2011.
19. S. Lurie et al. Comparison of maternal outcomes from primary cesarean section during the second compared with first stage of labor by indication for the operation. European Journal 44 of Obstetrics & Gynecology and Reproductive Biology 182 (2014) 43–47
20. Vitner D, Bleicher I, Levy E, Sloma R, Kadour-Peero E, Bart Y, Sagi S, Aviram A, Gonen R. Differences in outcomes between cesarean section in the second versus the first stages of labor. The Journal of Maternal-Fetal & Neonatal Medicine. 2019 Aug 3; 32(15):2539-42.
21. Cebekulu L, Buchmann EJ. Complications associated with cesarean section in the second stage of labor. International Journal of Gynecology & Obstetrics. 2006 Nov;95(2):110-4.
22. Pergialiotis V, Vlachos DG, Rodolakis A, Haidopoulos D, Thomakos N, Vlachos GD. First versus second stage C/S maternal and neonatal morbidity: a systematic review and meta-analysis. European Journal of Obstetrics & Gynecology and Reproductive Biology. 2014 Apr 1;175:15-24.
23. Thuillier C, Roy S, Peyronnet V, et al. Impact of recommended changes in labor management for prevention of the primary cesarean delivery. Am J Obstet Gynecol 2018; 218:341.e1-9.
24. Belay T, Yusuf L, Negash S. A comparative study on first stage versus second stage caesarean section on maternal and perinatal outcome. Ethiopian medical journal. 2014 Jan 1; 52(1):1-8.

For peer review only

For peer review only - http://bmjopen.bmj.com/site/about/guidelines.xhtml

25. Radha P, Shephali T, Muhammad Fairuz AR, Tee CS. Maternal and perinatal morbidity after caesarean delivery at full cervical dilatation. Singapore medical journal. 2012 Oct 1; 53(10):655.

26. Malachi Ochieng Arunda, Anette Agardh & Benedict Oppong Asamoah (2020) Cesarean delivery and associated socioeconomic factors and neonatal survival outcome in Kenya and Tanzania: analysis of national survey data, Global Health Action, 13:1, 1748403, DOI: 10.1080/16549716.2020.1748403

27. Epiu et al. Health and sustainable development; strengthening peri-operative care in low-income countries to improve maternal and neonatal outcomes. Reproductive Health (2018) 15:168

For peer review only

For peer review only - http://bmjopen.bmj.com/site/about/guidelines.xhtml

STROBE 2007 (v4) Statement—Checklist of items that should be included in reports of *cohort studies*

Section/Topic	Item #	Recommendation	Reported on page #
**Title and abstract**	1	(*a*) Indicate the study's design with a commonly used term in the title or the abstract	1 & 2
		(*b*) Provide in the abstract an informative and balanced summary of what was done and what was found	2
**Introduction**			
Background/rationale	2	Explain the scientific background and rationale for the investigation being reported	4 & 5
Objectives	3	State specific objectives, including any prespecified hypotheses	5
**Methods**			
Study design	4	Present key elements of study design early in the paper	6
Setting	5	Describe the setting, locations, and relevant dates, including periods of recruitment, exposure, follow-up, and data collection	5 & 6
Participants	6	(*a*) Give the eligibility criteria, and the sources and methods of selection of participants. Describe methods of follow-up	5
		(*b*) For matched studies, give matching criteria and number of exposed and unexposed	n/a
Variables	7	Clearly define all outcomes, exposures, predictors, potential confounders, and effect modifiers. Give diagnostic criteria, if applicable	6
Data sources/ measurement	8*	For each variable of interest, give sources of data and details of methods of assessment (measurement). Describe comparability of assessment methods if there is more than one group	*5 & 6*
Bias	9	Describe any efforts to address potential sources of bias	5
Study size	10	Explain how the study size was arrived at	5
Quantitative variables	11	Explain how quantitative variables were handled in the analyses. If applicable, describe which groupings were chosen and why	6
Statistical methods	12	(*a*) Describe all statistical methods, including those used to control for confounding	6
		(*b*) Describe any methods used to examine subgroups and interactions	6
		(*c*) Explain how missing data were addressed	6
		(*d*) If applicable, explain how loss to follow-up was addressed	6
		(*e*) Describe any sensitivity analyses	6
**Results**			

For peer review only

Participants	13*	(a) Report numbers of individuals at each stage of study—eg numbers potentially eligible, examined for eligibility, confirmed eligible, included in the study, completing follow-up, and analysed	6 & 7
		(b) Give reasons for non-participation at each stage	6
		(c) Consider use of a flow diagram	n/a
Descriptive data	14*	(a) Give characteristics of study participants (eg demographic, clinical, social) and information on exposures and potential confounders	7 & 8
		(b) Indicate number of participants with missing data for each variable of interest	n/a
		(c) Summarise follow-up time (eg, average and total amount)	n/a
Outcome data	15*	Report numbers of outcome events or summary measures over time	9 & 10
Main results	16	(*a*) Give unadjusted estimates and, if applicable, confounder-adjusted estimates and their precision (eg, 95% confidence interval). Make clear which confounders were adjusted for and why they were included	9 & 10
		(*b*) Report category boundaries when continuous variables were categorized	7, 8, 9
		(*c*) If relevant, consider translating estimates of relative risk into absolute risk for a meaningful time period	n/a
Other analyses	17	Report other analyses done—eg analyses of subgroups and interactions, and sensitivity analyses	n/a
**Discussion**			
Key results	18	Summarise key results with reference to study objectives	10, 11
**Limitations**			
Interpretation	20	Give a cautious overall interpretation of results considering objectives, limitations, multiplicity of analyses, results from similar studies, and other relevant evidence	12
Generalisability	21	Discuss the generalisability (external validity) of the study results	12
**Other information**			
Funding	22	Give the source of funding and the role of the funders for the present study and, if applicable, for the original study on which the present article is based	13, 14

*Give information separately for cases and controls in case-control studies and, if applicable, for exposed and unexposed groups in cohort and cross-sectional studies.

Note: An Explanation and Elaboration article discusses each checklist item and gives methodological background and published examples of transparent reporting. The STROBE checklist is best used in conjunction with this article (freely available on the Web sites of PLoS Medicine at http://www.plosmedicine.org/, Annals of Internal Medicine at http://www.annals.org/, and Epidemiology at http://www.epidem.com/). Information on the STROBE Initiative is available at www.strobe-statement.org.

For peer review only

Association between the stage of labor during cesarean delivery with adverse maternal and neonatal outcomes among referred mothers to tertiary centers in resource-limited settings

Journal:	*BMJ Open*
Manuscript ID	bmjopen-2023-077265.R1
Article Type:	Original research
Date Submitted by the Author:	20-Oct-2023
Complete List of Authors:	Zewdu, Dereje ; Wolkite University, Department of Anesthesia<br>Tantu, Temesgen ; Wolkite University, Obstetrics and Gynecology<br>Degemu, Fikretsion; Wolkite University, Department of Pediatrics and Child Health<br>Abdlwehab, Mukerem; Wolkite University, Department of Pediatrics and Child Health
**Primary Subject Heading**:	Obstetrics and gynaecology
Secondary Subject Heading:	Anaesthesia, Epidemiology
Keywords:	OBSTETRICS, Anaesthesia in obstetrics < ANAESTHETICS, NEONATOLOGY

SCHOLARONE™
Manuscripts

I, the Submitting Author has the right to grant and does grant on behalf of all authors of the Work (as defined in the below author licence), an exclusive licence and/or a non-exclusive licence for contributions from authors who are: i) UK Crown employees; ii) where BMJ has agreed a CC-BY licence shall apply, and/or iii) in accordance with the terms applicable for US Federal Government officers or employees acting as part of their official duties; on a worldwide, perpetual, irrevocable, royalty-free basis to BMJ Publishing Group Ltd ("BMJ") its licensees and where the relevant Journal is co-owned by BMJ to the co-owners of the Journal, to publish the Work in this journal and any other BMJ products and to exploit all rights, as set out in our licence.

The Submitting Author accepts and understands that any supply made under these terms is made by BMJ to the Submitting Author unless you are acting as an employee on behalf of your employer or a postgraduate student of an affiliated institution which is paying any applicable article publishing charge ("APC") for Open Access articles. Where the Submitting Author wishes to make the Work available on an Open Access basis (and intends to pay the relevant APC), the terms of reuse of such Open Access shall be governed by a Creative Commons licence – details of these licences and which Creative Commons licence will apply to this Work are set out in our licence referred to above.

Other than as permitted in any relevant BMJ Author's Self Archiving Policies, I confirm this Work has not been accepted for publication elsewhere, is not being considered for publication elsewhere and does not duplicate material already published. I confirm all authors consent to publication of this Work and authorise the granting of this licence.

For peer review only

Association between the stage of labor during cesarean delivery with adverse maternal and neonatal outcomes among referred mothers to tertiary centers in resource-limited settings

Dereje Zewdu[1]*, Temesgen Tantu[2], Fikretsion Degemu[3], Mukerem Abdlwehab[3]

[1]Department of Anesthesia, College of Medicine and Health Science, Wolkite University, Wolkite, Ethiopia

[2]Department of Obstetrics and Gynecology, College of Medicine and Health Science, Wolkite University, Wolkite, Ethiopia

[3]Department of Pediatrics and Child Health, College of Medicine and Health Science, Wolkite University, Wolkite, Ethiopia

*Corresponding author

Name: Dereje Zewdu

Email: Derejezewdu1529@gmail.com

Phone Number: +251 9240 4631

For peer review only

Abstract

Objective: Although the cesarean deliveries (CD) rate has substantially increased, little is known about its impacts when performed in the first and second stages of labor on fetomaternal outcomes, especially among referred mothers. Thus, this study aimed to investigate the association between cesarean deliveries (CD) performed during the first and second stages of labor and poor maternal and neonatal outcomes among mothers referred to tertiary centers.

Setting: This retrospective cohort study analyzed medical records of mother-infant pairs from September 2020 to May 2023 in Southern Ethiopia.

Participants: We retrospectively collected data from 848 participants who underwent emergency CD on a referral basis during the study period.

Primary outcome measure: The primary outcomes of interest were adverse maternal and neonatal outcomes. Data were analyzed using descriptive and inferential statistics.

Results: Of the 848 Cesarean deliveries, 722 (85.2%) and 126 (14.8%) were performed at the first and second stages of labor, respectively. Cesarean sections performed at the second stage were higher with nulliparity, increased maternal age, and birth weight. Compared with the first-stage CD, the second-stage CD was associated with a significantly increased risk of adverse maternal (OR = 3.7, 95% CI 2.4–5.7) and neonatal outcomes (OR 2.0; 95% CI 1.3–2.9), including neonatal death.

Conclusion: Second-stage CDs have an increased risk of adverse maternal and neonatal outcomes. Strengthening and improving obstetric emergency surgical services and intensive neonatal care for those populations would help decrease the maternal and fetal negative consequences.

Keywords: Cesarean delivery, Stage of Labor, maternal adverse outcomes, neonatal adverse outcomes

For peer review only

Article summary

Strengths and limitations of this study

- The study evaluated the impact of second-stage CD on fetomaternal outcomes among referred mothers using a larger sample size.
- The study followed the maternal and neonatal characteristics from admission to hospital discharge.
- The study included 3-year data, which is relatively representative of the existing problems.
- The retrospective nature of the study design may result in variable selection bias.
- Extrapolating the findings to non-referred participants might be difficult.

For peer review only

Introduction

The cesarean delivery (CD) rate has significantly increased globally, representing one-third of total institutional deliveries in Ethiopia. [1, 2] Although CD is generally a life-saving procedure, it may lead to increased maternal and neonatal complications and healthcare expenses. [3, 4] In many low-income countries where equitable access to obstetric surgical services is limited, the patient frequently gets referred to nearby tertiary centers and receives delayed treatment, resulting in compromised maternal and neonatal outcomes. [5, 6]

It's interesting to note that increased access to cesarean delivery in resource-limited settings could help reduce the incidence of maternal and newborn morbidity and mortality. [7] Nonetheless, being referred to other centers for CD and delays in timely intervention contribute to the likelihood of unfavorable fetomaternal outcomes. [8] Despite these, the impact of the stage of labor during CD on maternal and neonatal adverse outcomes among those populations has not been well-established. While clinicians carried out many CD procedures during the first stage of labor with fewer undesirable outcomes for both the mother and the newborn, the second-stage CD is technically more difficult and poses serious consequences. [9, 10]

Previous studies demonstrated inconclusive and conflicting results on the association between CD at the stage of labor and fetomaternal outcomes. Some studies have found a significant correlation between CD performed at the second stage of labor and adverse maternal effects. [11-18] On the contrary, others found no association between second-stage CD and poor maternal outcomes. [19-21] Poor maternal outcomes among CD performed at the second stage of labor include increased blood loss, uterine atony, need for blood transfusion, and perioperative complications.

Concerning neonatal outcomes, one study reported serious neonatal complications are common in the CD performed at the first stage of labor. [10] Nevertheless, other studies found higher poor outcomes in second-stage CD [13, 14, 21, and 22] and no association between these measurements. [15, 17, 23-25] Neonatal adverse outcomes include a low 5th-minute Apgar score, increased need for NICU admission, and neonatal death.

As far as our search, no other studies have compared the association between CD performed at the first and second stage of labor on their neonatal and maternal outcomes among referred mothers to tertiary centers. For practitioners to pinpoint the existing clinical gap and utilize the

For peer review only

findings to improve clinical practice, it would be beneficial to understand the connections between CD carried out at the first and second stages of labor and undesirable newborn and mother outcomes.

Therefore, we aimed to investigate the association between CD performed at the first and second stage of labor with adverse maternal and neonatal outcomes among mothers referred to Wolkite University Referral and Teaching Hospital in Southern Ethiopia. The findings of this study provide information for clinicians, researchers, and policymakers to maximize the quality of perioperative obstetric and intensive neonatal care and decrease second-stage CD-related fetomaternal complications.

Methods

Study population and study design

This retrospective cohort study included all consecutive women who underwent primary cesarean delivery for singleton term pregnancy between September 2020 and May 2023 at Wolkite University Specialized and Teaching Hospital in Ethiopia.

We have conducted the study per the Declaration of Helsinki Ethical Principles for Medical Research involving human subjects protocol. The study was approved by the Wolkite University Ethical Review Board and informed written consent was waived due to the retrospective nature of the study design.

We included a total of 848 consecutive primary cesarean deliveries performed for referred mothers during the study period for final analysis. Exclusion criteria were as follows: mothers with multiple gestation, preterm birth, intrauterine growth restriction, fetal anomalies, and incomplete documentation.

Data collection

We retrospectively collected data from the maternal and neonatal medical records, including maternal and neonatal baseline characteristics, perioperative data, and adverse maternal and neonatal outcomes of all study participants using a checklist adapted from prior studies. [26]

For peer review only

The maternal and neonatal baseline characteristics included maternal age, body mass index, parity, residency area, educational status, onset and duration of labor, gestational age, birth weight, and fetal sex. Perioperative maternal and neonatal characteristics included skin incision to delivery time, operation duration, estimated blood loss, uterine atony, unintentional uterine tear extension, uterine artery ligation, need for blood transfusion, Ureteral/bowel injury, perioperative hysterectomy, need for ICU admission, and maternal death, as well as 5th minute Apgar score, need for NICU admission, and neonatal death.

Study outcomes

The primary outcomes of interest were adverse maternal and neonatal outcomes. The maternal outcomes included one or more of the following: perioperative hemorrhage, uterine artery ligation, unintentional uterine extension, ureteral or bowel injury, peripartum hysterectomy, and maternal death. Neonatal adverse outcomes included one or more of the following: 5th minute Apgar score < 7, need for NICU admission, and neonatal death.

Statistical analysis

We entered, coded, and analyzed the data using SPSS (Statistical Package for the Social Sciences for Windows version 26.0, SPSS Inc., and Chicago, Illinois, USA). We used the student t-test test for numerical variables and described them as mean ± SD.

We presented the categorical data as frequency and percentage, and the association between independent and dependent variables was analyzed using the Chi-square and Fisher exact tests for statistical differences between groups. Logistic regression analysis examined the impacts of CD performed at the first and second stages of labor on maternal and neonatal adverse outcomes. Results displayed as an odds ratio with a 95% confidence interval (CI) and a p-value less than 0.05 are considered statistically significant.

Patient and public involvement

None

Results

For peer review only

Maternal and neonatal baseline characteristics

Of 897 women who underwent primary cesarean section performed during the study period, about 848 (94.5%) were included for final analysis. Of these, 722 (85.1%) cesarean deliveries were in the first stage and 126 ((14.9%) in the second stage of labor. While education, referral status, the labor onset, and fetal sex between CD performed at the first and second stages were comparable, nulliparity, the onset of labor to delivery time, and birthweight were significantly higher in the second-stage CD [58.7% vs. 35.7%; p-value < 0.001], [14.8 ± 10.4 vs. 10.6 ± 7.1; p-value < 0.001], and [3298.1 ± 338.3 vs. 3444.1 ± 322.3; p-value < 0.001], respectively as shown (Table 1).

Table 1: Maternal and neonatal baseline characteristics between CD performed at the first and second stages of labor

Variables	CD at 1st stage of labor (n=722)	CD at 2nd stage of labor (n=126)	p-value
Maternal age, year, mean ± SD	26.5 ± 4.8	24.9 ± 4.5	**< 0.001**
BMI, Kg/m^2, mean ± SD	27.9 ± 2.5	28.6 ± 2.5	0.004
Nulliparity, n (%)			**< 0.001**
Yes	258 (35.7)	74 (58.7)	
No	464 (64.3)	52 (41.3)	
Educational status, n (%)			0.17
Literate	588 (81.4)	96 (76.2)	
Illiterate	134 (18.6)	30 (23.8)	
Referral status, n (%)			0.62
Rural	338 (46.8)	62 (49.2)	
Urban	384 (53.2)	64 (50.8)	
The onset of labor, n (%)			0.65
Spontaneous	662 (91.7)	114 (90.5)	
Induced	60 (8.3)	12 (9.5)	
Labor onset to delivery, hours, mean ± SD	10.6 ± 7.1	14.8 ± 10.4	**< 0.001**

For peer review only

For peer review only - http://bmjopen.bmj.com/site/about/guidelines.xhtml

**Neonatal**			
GA at delivery, weeks, mean ± SD	38.6 ± 1.3	38.7 ± 1.5	0.54
Birthweight at delivery, gram, mean ± SD	3298.1 ± 338.3	3444.1 ± 322.3	**< 0.001**
Fetal sex, n (%)			0.9
Male	375 (51.9)	66 52.4)	
Female	347 (48.1)	60 (47.6)	

BMI: body mass index, SD: standard deviation, P < 0.05 is considered significant.

Perioperative maternal and neonatal characteristics

maternal perioperative characteristics, as shown in (Table 2), the CD performed at the second stage of labor was significantly associated with prolonged skin incision-to-delivery time, operation time, postpartum hemorrhage, uterine atony, uterine artery ligation, need for blood transfusion, unintentional uterine tear extension, perioperative hysterectomy, need for ICU admission, and even ureteral/bowel injury ($p < 0.001$, p < < 0.001, $p < 0.001$, $p < 0.001$, $p = 0.002$, $p = 0.008$, $p < 0.001$, $p = 0.016$, $p = 0.026$, $p = 0.036$, respectively).

Similarly, neonatal outcomes, including 5th minute Apgar score < 7, need for NICU admission, and neonatal death were significantly higher in CD performed at the second stage than in the first stage of labor (22.2% vs. 8.3%; $p < 0.001$, 34.9% vs. 24.6%; $p < 0.001$, 14.3% vs. 3.6%; $p < 0.001$), respectively as shown in (Table 2).

Table 2: Perioperative maternal and neonatal characteristics between CD performed at the first and second stages of labor

Variables	CD at the first stage of labor (n=722)	CD at the second stage of labor (n=126)	p-value
Skin incision to delivery time, minutes	6.1 ± 1.6	7.7 ± 2.2	**< 0.001**
Duration of operation, minutes	41.8 ± 20.1	49.4 ± 26.1	**< 0.001**
Duration of operation >1 hour, n (%)			**< 0.001**
Yes	122 (16.9)	42 (33.4)	
No	600 (83.1)	84 (66.6)	

For peer review only

Estimated blood loss, ml	464.5 ± 342.2	599.2 ± 539.2	**< 0.001**
Perioperative hemorrhage (> 1000ml), n (%)			**< 0.001**
Yes	44 (6.1)	20 (15.9)	
No	678 (93.9)	106 (84.1)	
Uterine atony, n (%)			**< 0.001**
Yes	34 (4.7)	18 (14.3)	
No	688 (95.3)	108 (85.7)	
Uterine artery ligation, n (%)	18 (2.5)	10 (7.9)	0.002
Blood transfusion, n (%)			0.008
Yes	22 (3)	10 (7.9)	
No	700 (97)	116 (92.1)	
Unintentional uterine tear extension, n (%)	4 (0.5)	7 (5.7)	**< 0.001**
Perioperative hysterectomy, n (%)	8 (1.1)	5 (4.1)	0.016
ICU admission, n (%)	12 (1.6)	6 (4.9)	0.026
Ureteral/bowel injury, n (%)	4 (0.5)	3 (2.4)	0.036[a]
Maternal death, n (%)	3 (0.4)	2 (1.6)	0.08
**Neonatal**			
5th minute Apgar score < 7, n (%)			**< 0.001**
Yes	60 (8.3)	28 (22.2)	
No	662 (91.7)	98 (77.8)	
Need for NICU admission, n (%)			**< 0.001**
Yes	178 (24.6)	44 (34.9)	
No	544 (75.4)	82 (65.1)	
Neonatal death, n (%)			**< 0.001**
Yes	26 (3.6)	18 (14.3)	
No	696 (96.4)	108 (85.7)	

NICU: neonatal intensive care unit, ICU: intensive care unit, CD: cesarean delivery, [a:] Fisher exacts test

For peer review only

Association between CD performed at the first and second stage of labor with adverse maternal and neonatal outcomes

Logistic regression analysis showed that the likelihood of developing adverse maternal outcomes after CD performed at the second stage of labor was 3.7 times (OR = 3.7, 95% CI 2.4 – 5.7) higher than the first-stage CD. Similarly, second-stage CD increases the risk of developing adverse neonatal outcomes 2-fold (OR 2.0; 95% CI 1.3 – 2.9) compared to CD performed at the first stage of labor, as shown in (Table 3)

Table 3: Association of adverse maternal and neonatal outcomes with CD performed at the first and second stages of labor

Outcomes	CD at the first stage of labor (n=722)	CD at the second stage of labor (n=126)	Odd ratios (95% CI)	p-value
Adverse maternal outcomes				**<0.001**
Yes	88 (12.2)	43 (34.1)	3.7 (2.4 – 5.7)	
No	634 (87.8)	88 (65.9)	1	
Adverse neonatal outcomes				**<0.001**
Yes	198 (27.4)	54 (42.8)	2.0 (1.3 – 2.9)	
No	524 (72.6)	72 (57.2)	1	

CD: cesarean delivery, CI: confidence interval

Discussion

In this retrospective study involving 848 women who underwent emergency cesarean delivery at a referral hospital in Southern Ethiopia, we compared maternal and neonatal adverse outcomes of CD performed during the first and second stages of labor. Our main findings were that CD performed at the second stage was associated with an increased risk of maternal and neonatal adverse outcomes compared to CD performed at the first stage of labor.

For peer review only

Increased adverse maternal outcomes included longer operation times, estimated blood loss or postpartum hemorrhage, uterine atony, and uterine artery ligation procedures, suggesting that CD at the second stage might be technically challenging and associated with higher odds of complications. [9, 10] This is likely due to the engagement of the fetal head in the pelvis, the difficulty in delineating the bladder, and the larger infants that are perhaps more difficult to remove from the uterus. [11, 12]

The increased amount of blood loss because of uterine atony and unintentional uterine extensions is frequent in the second stage CD, as revealed in the present and previous studies. [13, 14] Other studies also showed that unintended uterine extension was associated with longer operation time, more blood loss, postpartum anemia, and an increased need for blood transfusion. [15, 16]

We believe that the extended labor duration and more substantial uterine fatigue may be the reason for the increase in uterine atony incidence in the second stage CD. [17] Our study also showed because of the increased amount of blood loss and uterine atony, an increased need for blood transfusion and uterine artery ligation procedures are more frequent in second-stage CD, in line with previous reports. [18] Therefore, it is crucial to consider effective uterotonic agents, leveraging the expertise of experienced obstetricians, and continual monitoring to lessen maternal morbidity in these scenarios.

Contradicting our findings, a few studies [19-21] reported no association between second-stage CD and more blood loss, uterine atony, and other poor maternal outcomes. These discrepancies may occur because of the variations in the study population recruited for analysis (urgency of the surgery, indications for CD, and delayed presentation in their referral status), fluctuations in sample size and study design, and disparities in the standard of obstetric and neonatal care between the study settings. To that end, the current study included referred mothers from local health centers for primary CDs only, which increases the risk of fetomaternal complications.

Our study also found that peripartum hysterectomy, the need for ICU admission, and urethral/bowel injury were higher among mothers who underwent CD at the second stage of labor; however, the small number of those complications are insufficient to conclude.

For peer review only

On the other hand, we observed a significantly higher association between CD performed at the second stage of labor and adverse neonatal outcomes, including a 5th-minute Apgar score < 7, the need for NICU admission, and neonatal death than CD performed at the first stage of labor. In agreement with our findings, previous studies [13, 22] showed a significant association between the second stage of CD and neonatal morbidity, including an increased incidence of 5th minute Apgar score < 7 and higher NICU admission rates, but failed to show an association with neonatal death. In disagreement, our findings show that the CD performed at the second stage of labor is not only associated with neonatal morbidity but also with neonatal mortality, in line with other studies. [14, 22, 26]

On the contrary, some studies even observed the insignificant impact of CD performed at the second stage of labor on adverse neonatal outcomes. [15, 17, 23-25] These might indicate that the mode of delivery being emergency cesarean section, referred from other centers, and the poor quality of neonatal intensive care units in our study settings could justify the observed difference, suggesting the need for improving intensive neonatal care in resource-limited settings. Also, obstetric surgical access is limited in African countries and is responsible for increasing referrals to tertiary centers; when performed, it is often too late to reduce perinatal deaths and is associated with poorer neonatal outcomes, especially during emergency CD. [8]

Thus, providing equitable obstetric surgical access and strengthening obstetric surgical and intensive neonatal care in low-income countries need immediate attention to improve maternal and perinatal outcomes. [26-29]

Strength and limitations

The strength of our study is that only referred mothers are included as a source population, which allows a comparison of the obstetric outcomes of these underserved populations to exemplify the existing problems. However, the retrospective nature of the study design precludes the inclusion of the most significant covariates, which might affect the impact of the stage of labor on the outcome variables. We also relied on data retrieved from the medical records of a single institution, making it difficult to extrapolate the findings to the non-referred mothers in the study setting. Furthermore, because of substandard overall NICU care and poor documentation in our hospital, we failed to include additional adverse neonatal outcomes, such as septicemia, intubation, seizures, and fetal injuries. Therefore, a further prospective study, including large

For peer review only

sample size, multiple study hospitals, and various neonatal outcomes, is needed to determine the generalizability of the findings to the entire study population. Despite these limitations, obstetric care providers should consider the impact of second-stage cesarean deliveries and provide effective management to lower the burden of such cases.

Conclusion

CS performed at the second stage of labor among referred mothers is associated with an increased risk of adverse maternal and neonatal outcomes. Thus, strengthening and improving obstetric emergency surgical services and intensive neonatal care for those populations would help reduce poor maternal and fetal outcomes. Further investigation may also require improving obstetric and neonatal intensive care.

Acknowledgments

The authors acknowledge Wolkite University's Specialized and Teaching Hospital Management, data collectors, and medical record office staff for their invaluable support.

Contributors

DZ, TT, FD, and MA conceived and designed the study. DZ and TT performed the data collection, data analysis, and initial draft manuscript. DZ and MA reviewed the manuscript critically. All authors read and approved the final manuscript.

Funding

The authors have not declared a specific grant for this research from any funding agency in the public, commercial, or not-for-profit sectors.

Competing interests

None declared.

Patient consent

Not required.

For peer review only

Ethics approval

Wolkite University Ethical Review Board (ERB) provided the ethical clearance for the study, and written consent was exempted by ERB because of the retrospective nature of the study design.

Provenance and peer review

Not commissioned; externally peer-reviewed.

Data sharing statement

All data supporting our findings will be shared on request.

Contact

Derejezewdu1529@gmail.com

For peer review only

References

1. Betrán AP, Ye J, Moller AB, Zhang J, Gülmezoglu AM, Torloni MR. The increasing trend in cesarean section rates: global, regional and national estimates: 1990-2014. PloS one. 2016 Feb 5; 11(2):e0148343.
2. Gedefaw G, Demis A, Alemnew B, Wondmieneh A, Getie A, Waltengus F. Prevalence, indications, and outcomes of cesarean section deliveries in Ethiopia: a systematic review and meta-analysis. Patient safety in surgery. 2020 Dec; 14(1):1-0.
3. Elias S, Wolde Z, Tantu T, Gunta M, Zewudu D. Determinants of early neonatal outcomes after emergency cesarean delivery at Hawassa University comprehensive specialised hospital, Hawassa, Ethiopia. Plos one. 2022 Mar 24; 17(3):e0263837.
4. Zewdu D, Tantu T. Incidence and predictors of severe postpartum hemorrhage after cesarean delivery in South Central Ethiopia: a retrospective cohort study. Scientific Reports. 2023 Mar 3; 13(1):3635.
5. Sobhy S, Arroyo-Manzano D, Murugesu N, Karthikeyan G, Kumar V, Kaur I, Fernandez E, Gundabattula SR, Betran AP, Khan K, Zamora J. Maternal and perinatal mortality and complications associated with caesarean section in low-income and middle-income countries: a systematic review and meta-analysis. The Lancet. 2019 May 11; 393(10184):1973-82.
6. Ebener S, Stenberg K, Brun M, Monet JP, Ray N, Sobel HL, Roos N, Gault P, Conlon CM, Bailey P, Moran AC. Proposing standardised geographical indicators of physical access to emergency obstetric and newborn care in low-income and middle-income countries. BMJ global health. 2019 Jun 1; 4(Suppl 5):e000778.
7. Irani M, Deering S. Challenges affecting access to cesarean delivery and strategies to overcome them in low-income countries. International Journal of Gynecology & Obstetrics. 2015 Oct 1; 131(1):30-4.
8. Shah A, Fawole B, M'imunya JM, Amokrane F, Nafiou I, Wolomby JJ, Mugerwa K, Neves I, Nguti R, Kublickas M, Mathai M. Cesarean delivery outcomes from the WHO global survey on maternal and perinatal health in Africa. International Journal of Gynecology & Obstetrics. 2009 Dec 1; 107(3):191-7.
9. Dalvi SA. Difficult deliveries in cesarean section. the Journal of Obstetrics and Gynecology of India. 2018 Oct; 68(5):344-8.

For peer review only

10. Lipschuetz M, Guedalia J, Cohen SM, Lewkowicz AA, Amsalem H, Karavani G, Yagel S, Unger R. Unplanned cesarean delivery in the second stage of labor holds higher odds of complications than in the first stage, while similar in primiparas and multiparas. Fetal Diagnosis and Therapy. 2020; 47(7):565-71.

11. Jennifer B Manning, Mary C Tolecher, Edwin C, et al: Delivery of an Impacted fetal head during Caesarian—A literature review and proposed management algorithm. Obstet Gynecol Surv. 2015; 70(11):719–24.

12. Asıcıoglu O, Güngördük K, Yildirim G, Asıcıoglu BB, Güngördük ÖÇ, Ark C, Günay T, Yenigül N. Second-stage vs first-stage caesarean delivery: comparison of maternal and perinatal outcomes. Journal of Obstetrics and Gynaecology. 2014 Oct 1; 34(7):598-604.

13. Sinha S, Malik S, Dixit M. First versus second stage caesarean section: a comparison of maternal and neonatal outcomes. International Journal of Reproduction, Contraception, Obstetrics and Gynecology. 2017 Aug 1; 6(8):3477-81.

14. Seal SL, Kamilya G, Mukherji J, Bhattacharyya SK, De A, Hazra A. Outcome in second- versus first-stage cesarean delivery in a teaching institution in eastern India. American Journal of Perinatology. 2010 Jun; 27(06):507-12.

15. Sung JF, Daniels KI, Brodzinsky L, El-Sayed YY, Caughey AB, Lyell DJ. Cesarean delivery outcomes after a prolonged second stage of labor. American Journal of Obstetrics and Gynecology. 2007 Sep 1; 197(3):306-e1.

16. Renana Wilkof-Segev, Amir Naeh, Sivan Barda, Mordechai Hallak & Rinat Gabbay-Benziv (2023) Unintended uterine extension at the time of cesarean delivery – risk factors and associated adverse maternal and neonatal outcomes, The Journal of Maternal-Fetal & Neonatal Medicine, 36:1, 2204997, DOI: 10.1080/14767058.2023.2204997

17. Alexander JM, Leveno KJ, Rouse DJ, Landon MB, Gilbert S, Spong CY, Varner MW, Moawad AH, Caritis SN, Harper M, Wapner RJ. Comparison of maternal and infant outcomes from primary cesarean delivery during the second compared with first stage of labor. Obstetrics & Gynecology. 2007 Apr 1; 109(4):917-21.

18. Sucak A, Çelen Ş, Akbaba E, Soysal S, Moraloglu O, Danışman N. Comparison of nulliparas undergoing cesarean section in first and second stages of labour: a prospective study in a tertiary teaching hospital. Obstetrics and gynecology international. 2011 Jan 1; 2011.

For peer review only

19. S. Lurie et al. Comparison of maternal outcomes from primary cesarean section during the second compared with first stage of labor by indication for the operation. European Journal 44 of Obstetrics & Gynecology and Reproductive Biology 182 (2014) 43–47
20. Vitner D, Bleicher I, Levy E, Sloma R, Kadour-Peero E, Bart Y, Sagi S, Aviram A, Gonen R. Differences in outcomes between cesarean section in the second versus the first stages of labor. The Journal of Maternal-Fetal & Neonatal Medicine. 2019 Aug 3; 32(15):2539-42.
21. Cebekulu L, Buchmann EJ. Complications associated with cesarean section in the second stage of labor. International Journal of Gynecology & Obstetrics. 2006 Nov; 95(2):110-4.
22. Pergialiotis V, Vlachos DG, Rodolakis A, Haidopoulos D, Thomakos N, Vlachos GD. First versus second stage C/S maternal and neonatal morbidity: a systematic review and meta-analysis. European Journal of Obstetrics & Gynecology and Reproductive Biology. 2014 Apr 1; 175:15-24.
23. Thuillier C, Roy S, Peyronnet V, et al. Impact of recommended changes in labor management for prevention of the primary cesarean delivery. Am J Obstet Gynecol 2018; 218:341.e1-9.
24. Belay T, Yusuf L, Negash S. A comparative study on first stage versus second stage caesarean section on maternal and perinatal outcome. Ethiopian medical journal. 2014 Jan 1; 52(1):1-8.
25. Radha P, Shephali T, Muhammad Fairuz AR, Tee CS. Maternal and perinatal morbidity after caesarean delivery at full cervical dilatation. Singapore medical journal. 2012 Oct 1; 53(10):655.
26. Zewdu D, Tantu T, Degemu F, Hawlet M, Dejene N and Asefa E (2023) Exploring factors influencing skin incision to the delivery time and their impact on neonatal outcomes among emergency cesarean deliveries indicated for non-reassured fetal heart rate status. Front. Pediatr. 11:1224508. doi: 10.3389/fped.2023.1224508
27. Malachi Ochieng Arunda, Anette Agardh & Benedict Oppong Asamoah (2020) Cesarean delivery and associated socioeconomic factors and neonatal survival outcome in Kenya and Tanzania: analysis of national survey data, Global Health Action, 13:1, 1748403, DOI: 10.1080/16549716.2020.1748403

For peer review only

28. Epiu et al. Health and sustainable development; strengthening peri-operative care in low-income countries to improve maternal and neonatal outcomes. Reproductive Health (2018) 15:168

29. D. Zewdu, T. Tantu, R. Ali, et al. Prevalence and predictors of postpartum anemia after caesarean delivery in Ethiopia: A retrospective analysis of risk factors. Midwifery 123 (2023) 103707

For peer review only

STROBE 2007 (v4) Statement—Checklist of items that should be included in reports of *cohort studies*

Section/Topic	Item #	Recommendation	Reported on page #
**Title and abstract**	1	(*a*) Indicate the study's design with a commonly used term in the title or the abstract	1 & 2
		(*b*) Provide in the abstract an informative and balanced summary of what was done and what was found	2
**Introduction**			
Background/rationale	2	Explain the scientific background and rationale for the investigation being reported	4 & 5
Objectives	3	State specific objectives, including any prespecified hypotheses	5
**Methods**			
Study design	4	Present key elements of study design early in the paper	6
Setting	5	Describe the setting, locations, and relevant dates, including periods of recruitment, exposure, follow-up, and data collection	5 & 6
Participants	6	(*a*) Give the eligibility criteria, and the sources and methods of selection of participants. Describe methods of follow-up	5
		(*b*) For matched studies, give matching criteria and number of exposed and unexposed	n/a
Variables	7	Clearly define all outcomes, exposures, predictors, potential confounders, and effect modifiers. Give diagnostic criteria, if applicable	6
Data sources/ measurement	8*	For each variable of interest, give sources of data and details of methods of assessment (measurement). Describe comparability of assessment methods if there is more than one group	*5 & 6*
Bias	9	Describe any efforts to address potential sources of bias	5
Study size	10	Explain how the study size was arrived at	5
Quantitative variables	11	Explain how quantitative variables were handled in the analyses. If applicable, describe which groupings were chosen and why	6
Statistical methods	12	(*a*) Describe all statistical methods, including those used to control for confounding	6
		(*b*) Describe any methods used to examine subgroups and interactions	6
		(*c*) Explain how missing data were addressed	6
		(*d*) If applicable, explain how loss to follow-up was addressed	6
		(*e*) Describe any sensitivity analyses	6
**Results**			

For peer review only

Participants	13*	(a) Report numbers of individuals at each stage of study—eg numbers potentially eligible, examined for eligibility, confirmed eligible, included in the study, completing follow-up, and analysed	6 & 7
		(b) Give reasons for non-participation at each stage	6
		(c) Consider use of a flow diagram	n/a
Descriptive data	14*	(a) Give characteristics of study participants (eg demographic, clinical, social) and information on exposures and potential confounders	7 & 8
		(b) Indicate number of participants with missing data for each variable of interest	n/a
		(c) Summarise follow-up time (eg, average and total amount)	n/a
Outcome data	15*	Report numbers of outcome events or summary measures over time	9 & 10
Main results	16	(*a*) Give unadjusted estimates and, if applicable, confounder-adjusted estimates and their precision (eg, 95% confidence interval). Make clear which confounders were adjusted for and why they were included	9 & 10
		(*b*) Report category boundaries when continuous variables were categorized	7, 8, 9
		(*c*) If relevant, consider translating estimates of relative risk into absolute risk for a meaningful time period	n/a
Other analyses	17	Report other analyses done—eg analyses of subgroups and interactions, and sensitivity analyses	n/a
**Discussion**			
Key results	18	Summarise key results with reference to study objectives	10, 11
**Limitations**			
Interpretation	20	Give a cautious overall interpretation of results considering objectives, limitations, multiplicity of analyses, results from similar studies, and other relevant evidence	12
Generalisability	21	Discuss the generalisability (external validity) of the study results	12
**Other information**			
Funding	22	Give the source of funding and the role of the funders for the present study and, if applicable, for the original study on which the present article is based	13, 14

*Give information separately for cases and controls in case-control studies and, if applicable, for exposed and unexposed groups in cohort and cross-sectional studies.

Note: An Explanation and Elaboration article discusses each checklist item and gives methodological background and published examples of transparent reporting. The STROBE checklist is best used in conjunction with this article (freely available on the Web sites of PLoS Medicine at http://www.plosmedicine.org/, Annals of Internal Medicine at http://www.annals.org/, and Epidemiology at http://www.epidem.com/). Information on the STROBE Initiative is available at www.strobe-statement.org.

For peer review only
